# The Remains of the Day in Dissociative Amnesia

**DOI:** 10.3390/brainsci2020101

**Published:** 2012-04-10

**Authors:** Angelica Staniloiu, Hans J. Markowitsch

**Affiliations:** Physiological Psychology, University of Bielefeld, D-33501 Bielefeld, Germany; E-Mail: hjmarkowitsch@uni-bielefeld.de

**Keywords:** autonoetic consciousness, anoetic consciousness, priming, procedural memory, episodic-autobiographical memory, psychogenic amnesia

## Abstract

Memory is not a unity, but is divided along a content axis and a time axis, respectively. Along the content dimension, five long-term memory systems are described, according to their hierarchical ontogenetic and phylogenetic organization. These memory systems are assumed to be accompanied by different levels of consciousness. While encoding is based on a hierarchical arrangement of memory systems from procedural to episodic-autobiographical memory, retrieval allows independence in the sense that no matter how information is encoded, it can be retrieved in any memory system. Thus, we illustrate the relations between various long-term memory systems by reviewing the spectrum of abnormalities in mnemonic processing that may arise in the dissociative amnesia—a condition that is usually characterized by a retrieval blockade of episodic-autobiographical memories and occurs in the context of psychological trauma, without evidence of brain damage on conventional structural imaging. Furthermore, we comment on the functions of implicit memories in guiding and even adaptively molding the behavior of patients with dissociative amnesia and preserving, in the absence of autonoetic consciousness, the so-called “internal coherence of life”.

## 1. Introduction

Memory is a universal attribute of probably all animal species. The sense of personal identity of individuals (at least in individualistic, western societies) is strongly bound to their personal memories, in particular to those memories that have an episodic-autobiographical quality [1]. To have a good memory is considered to be an advantage for survival in animals. In humans, an intact episodic-autobiographical memory (EAM) system has also been posited to be a prerequisite for health and well-being [2]. EAM has been attributed retrospective, prospective, survival, directive and social functions [3]. The “loss” of memory—amnesia—was regarded as a major handicap for an individual and, in the past, frequently equated with dementia. In the context of increased life expectancy in western industrialized countries, media portrays an aging generation that is frightened of developing Alzheimer’s dementia, an illness perceived as being associated with losing memories and subsequently the personal identity. On the other hand, a close look at the literature about the life and experiences of great mnemonists suggests that the possession of extraordinary memory skills might not be perceived as a blessing by their owners, but rather as a big challenge to their “lightness of being” [4,5,6]. Jill Price, the case described by Parker *et al.* [6], for example, wrote in a letter about her memory: “Most have called it a gift but I call it a burden. I run my entire life through my head every day and it drives me crazy!!!” (p. 35). 

The need for a balance between forgetting and remembering has been pointed out since Ribot’s time [2]. In the same vein, William James [7] wrote: “If we remembered everything, we should on most occasions be as ill off as if we remembered nothing” (p. 262). The illness brought by remembering everything is metaphorically illustrated by Borges’ character, Funes, who reaches a state where he cannot act or even move any more [8]. Apart from pointing to the possible burden associated with excessive conscious remembering, other authors debated the proposed advantages associated with the existence of our highest ontogenetic memory system (EAM) and called into question the functions that have been attributed to this system over the years. With respect to the social function, it has been observed that most of the reading of the inner mental states of others does not involve accessing EAM representations, but rather relies on other “lower” forms of memory, described below [3,9]. Nevertheless, the connection between EAM and social cognition and behavior is a topic of increasing research interest in the light of neuroimaging findings showing overlaps between areas activated during tasks tapping into EAM, Theory-of-Mind, prospection and navigation (see [3] for review). The capacity for autonoetic consciousness, that is an integral feature of EAM, was traditionally viewed as playing a key role in maintaining the feeling of sameness over time, and more recently as being involved in preparing human beings for the demands of the future [10]. Autonoetic consciousness is the ability to reflect upon oneself and to distinguish oneself from others in social and biological environments. It is also seen as the capacity allowing adult humans “to mentally represent and to become aware of their protracted existence across subjective time” (p. 335) [11]. Some authors have, however, questioned the survival function of EAM by stating that autonoetic consciousness might bring with it the awareness of one’s own finitude, “a possibly fatal piece of knowledge, full understanding of which would preclude any motivation to survive” [12] (p. 762). Religion, faith, magic beliefs, false memories, motivated memory suppression and self deceptive mechanisms were all proposed to have (co-)evolved as a means of counteracting this possible fatal piece of knowledge accompanying a superior capacity for self awareness [12,13,14]. As Freud suggested in 1896, each act of recollection involves a process of re-transcription or “rearrangement in accordance to fresh circumstances” [15] (p. 207), and perhaps also an act of imagination [16]; subsequently our EAM’s are far from fulfilling the function of providing a reliable and faithful repository of the past. What remains from the day in our life narrative is the state- and context-dependent and -scaffolded product of several processes involving repression of memories and desires, cloaking of emotions, suppression, categorization, identification, displacement and non-deliberate falsification, which create a story by which we could at the end of the day live by in a societal and cultural context that cherishes individuation, autonomy, self determination and self control [17]. The dynamic changes that the EAM undergoes over time were posited by Conway [18] to serve the purpose of maintaining and supporting current aspects of the self and matching future goals that are coherent with one individual’s goals, self-image and system of beliefs. But is indeed EAM indispensable for our sense of personal identity and self [19]? And even if this were the case, how would the sense of preservation of a distinct, separate, severed personal identity contribute to one’s person’s well being in various cultures [20]? As we are going to illustrate below by resorting to examples from patients with dissociative amnesia, a sense of self that goes beyond one of a core self may survive in the absence of autonoetic consciousness, being grounded by lower memory systems, which enable human beings to preserve their habits and “internal coherence of life” [21]. Furthermore, the degree of perceived distress produced by the “loss” of memory in dissociative amnesia may be heavily colored by culturally-shaped models of personhood and past [22]. 

## 2. Amnesia and Dissociation

Borrowed from Greek [23] the word amnesia describes the most severe form of memory impairment and refers to an inability to learn new information or recall previously learned information. As the word shares a semantic kinship with the word “amnesty”, “amnesic” and “amnestic” are often used interchangeably, in spite of some authors objecting to this due to the juridical loading of the word “amnesty” [24]. The term “amnesia” is nowadays used to refer to a symptom of a disorder, a syndrome or a specific disease. In the latter case, its employment is congruent with the traditional view of amnesia as a memory impairment that occurs in an alert, responsive person in the absence of (or out of proportion in comparison to) other significant cognitive impairments, being subsequently restricted to specific disorders. These disorders are named amnesic [25] or amnestic [26] disorders. They have as a core feature a memory impairment that is not due to dementia, leads to impairment of functioning and represents a decline from a previously attained level of function. Based on their predominant etiological link amnes(t)ic disorders are categorized as being due to a general medical condition (e.g., neurological event), direct effects of substances (e.g., alcohol or benzodiazepines) persisting beyond the period of intoxication or withdrawal or psychological factors. The term psychogenic amnesia has traditionally been used to describe episodes of retrograde and/or anterograde (EAM) memory loss, which are precipitated by psychological stresses and occur in the absence of identifiable brain damage. Apart from psychogenic, other terminologies (such as hysterical, dissociative, functional or medically unexplained or mnestic block syndrome) have over the years been employed to capture the category of amnesic disorders without direct evidence of significant brain damage on conventional structural imaging techniques. In the current main official classifications of diseases [25,26], earlier diagnostic designations of hysterical or psychogenic amnesia are now predominantly subsumed under the diagnostic categories of dissociative disorders in DSM-IV-TR [26] and dissociative (conversion) disorders in ICD-10 [25]. However, these conditions also appear under other diagnostic subcategories, such as somatization disorder (in DSM-IV-TR [26] only), post-traumatic stress disorder and acute stress disorder (DSM-IV-TR [26] and ICD-10 [25]). Despite its gradual disappearance from the international nomenclatures of diseases, terms such as psychogenic of functional amnesia have remained alive in the psychological literature for a number of reasons. As opposed to the current ICD-10 [25] and DSM-IV-TR [26] diagnostic subcategory of dissociative amnesia, the term psychogenic amnesia is more comprehensive, being employed in the psychological literature to encompass more than one diagnostic entity. Although several cases of functional amnesia were found to occur in the background in psychological stress or trauma, alone or in combination with a co-occurring (mild) physical insult (such as a mild traumatic brain injury, mild electric shock, mild physical injury), there are case reports of functional amnesia where a clear-cut psychological, etiological mechanism could not be identified. This led de Renzi *et al.* [27] to propose that the concept of functional amnesia was a “more suitable term to classify patients whose memory disorders cannot be traced back to organic or psychological causes” (p. 788). The lack of clearly identifiable psychological triggers in some variants of functional amnesia may be explained by several factors. These include the memory disturbance for the stressful event due to amnesia: an impaired capacity for emotional awareness and processing in the face of ongoing or repeated stresses that seems to premorbidly characterize some patients with psychogenic amnesia, and in fact might predispose them to develop this condition [3], the possible involvement of mechanisms of kindling sensitization in the face of recurrent stresses, which may trigger an episode of illness after a seemingly minor stress, the so-called incubation-effect-of-life adversity [3]. 

Traditionally, memory problems in neurological patients were seen as global, leading to the label of “global amnesia”. Meanwhile it has been shown that memory deficits tend to predominantly affect certain kinds of memories, while sparing others and that, depending on their neural underpinnings, their occurrence may be accompanied by various clinical features. This reflects the existence of several memory systems, with different degrees of susceptibility to environmental insults and distinct, or partly distinct, neuroanatomical substrates. 

## 3. Memory Systems

Memory is not unitary, but is divided along chronological and content axes. Along the time axis, memory is categorized (classified in) into short-term and long-term memory. The term short-term memory has been employed to describe the online-holding of information such as telephone numbers. It has a limited capacity of a few bits (4–7) [28] and encompasses a time range of seconds to minutes. Any information that is not lost and exceeds the limited capacity of short-term and working memory is assigned to long-term memory stores. The time-related dichotomy of memory was further refined by the introduction of the term “working memory” [29]. As captured by its name, working memory refers to working with memory—this involves not only time limited online holding of new information, but also retrieving portions of old, already stored information. 

Further time-related categorizations of memories involve distinctions between old and new and anterograde and retrograde memories, respectively. The compromised ability to access information that happened before the memory-impairing incident corresponds to retrograde memory impairment, while anterograde memory impairment refers to the compromised capacity to long-term acquire new information after an incident. Anterograde amnesia can occur with minimal or no retrograde amnesia. Although traditional accounts of amnesia had led to the assumption that retrograde amnesia should be always accompanied by anterograde amnesia, cases of isolated retrograde amnesia have been reported after both neurological and psychological events. In some cases of isolated retrograde amnesia following a neurological event (severe traumatic brain injury), significant anterograde amnesia is present initially, but then resolves or becomes subtle [30]. The etiological mechanisms underlying isolated or disproportionate retrograde amnesia continue, however, to be a source of debate, with some authors arguing that even in cases with clear evidence of brain pathology, psychological factors might make a substantial contribution to the presence of residual, disproportionate retrograde EAM loss [31]. 

**Figure 1:**
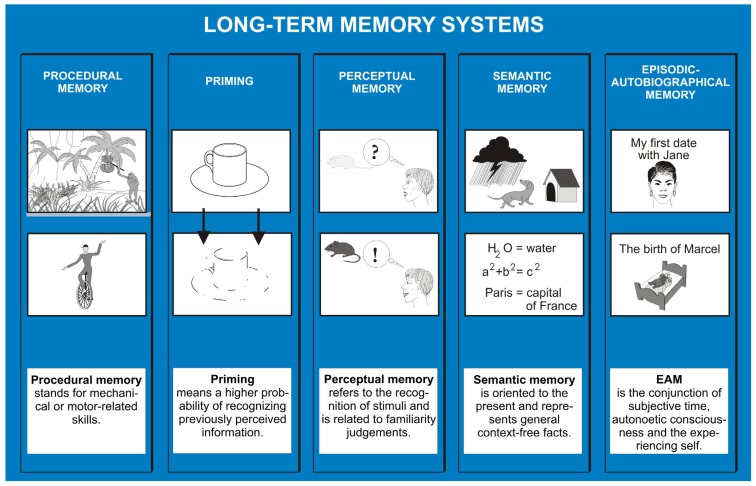
Sketch of the five long-term memory systems. Note that it is assumed that they develop both phylo- and ontogenetically from left to right.

The categorization of memory along the content dimension has emerged from data from patients with different types of memory impairments, corresponding to different types of brain lesions, neuroimaging studies of patients with memory impairments or normal subjects, animal memory research findings and human developmental studies. In the 1970s and 1980s, a revival of early conceptualizations of memory subdivisions [32] was proposed in the field of human memory research by Endel Tulving [33], and in the arena of animal research by Mortimer Mishkin [34]. Mishkin made the distinction between “habit” and “memory” systems; the habit system referred to procedures and routines, whereas the memory system was concerned with the acquisition of facts and relations between objects. Tulving initially differentiated between semantic and episodic memory, implying that semantic memory refers to general knowledge, and episodic memory to single episodes with a specific embedding in time and place. Later Tulving and other researchers expanded the categorization of memory systems, in particular by adding systems involving automatic, implicit and subconscious levels of processing—such as procedural memory and priming. Pfeifer and Bongard [35] remarked that memory has deep roots in the interaction of the body with the environment. This is also illustrated by Tulving’s [36] hierarchical model (Figure 1), which proposes that the development of memory systems starts with systems that involve processing of information (procedural memory, priming) that is devoid of the need for consciousness (anoetic), continues with conscious (noetic) systems—perceptual and then semantic memory—and apparently culminates with the emergence of the EAM system which requires autonoetic consciousness. As proposed by Vandekerckhove and Panksepp [37], and implied by the model of Tulving [36], higher forms of consciousness, such as autonoetic consciousness, are likely “embedded in the ancient affective soil of anoetic consciousness” (p. 1026). Furthermore, self consciousness is intertwined with bodily consciousness [37]. The terminologies autonoetic (“self-conscious”, or “self-aware”), noetic (“aware”) and anoetic (“not-aware”) were introduced by Tulving [38]. Anoetic consciousness is a capacity, probably common to all animals, to gain awareness of changes in stimulation. Noetic consciousness is a capacity, probably common to all animals, which can be used to gain awareness of objects, situations and states of the world, not present to senses [39]. Autonoetic consciousness entails a “sense of self in time and the ability to relive subjective experiences from the encoding context by mentally travelling back in time” [40] (p. 260).

Procedural memory refers to highly automated sensory-motor skills, which include complex motor acts like driving a car, riding a bike, or skiing. Priming describes the higher probability of (“automatically”) choosing a stimulus which was perceived earlier in the same or a similar manner. The “perceptual memory system” was identified as a legitimate distinct system relatively recently. In contrast to the priming and procedural memory systems, this system acts “consciously” (noetically), but on a presemantic level and relies on familiarity judgments. An example is the conscious (noetic) identification of an apple without hesitation, no matter what color it has or whether it is already half eaten or not. A relevant example is that of patients with semantic dementia, who in spite of losing capabilities for language and semantic memory, may still be able to distinguish, for example, an apple from a peach or pear, without the need to access semantic information, by accessing perceptual representations of information via the perceptual memory system.

The theoretical framework for understanding episodic memory and episodic memory systems has undergone refinements over the years. Several decades ago the term episodic could be applied to describe memory for laboratory stimuli with a specific embedding in time and place [33]; nowadays the episodic memory system is viewed as equivalent to the EAM system, and EAM is defined as the conjunction of subjective time, autonoetic consciousness and the experiencing self [36]. A main characteristic of EAM is considered to be mental time-traveling through subjective time from present to both past and future. This is enabled by chronesthesia, defined as “a form of consciousness that allows individuals to think about the subjective time in which they live and that makes it possible for them to mentally travel in such time” [10] (p. 22357). Mental time traveling through subjective time is one of the last features of EAM that becomes fully functional, but the first one to be degraded by age-related memory changes and most amnesic conditions [3]. Although the designations “autobiographical” and “episodic” are sometimes used interchangeably, not all autobiographical memories have an episodic quality. A distinction is nowadays made between autobiographical-episodic and autobiographical-semantic memory. The latter refers to personal knowledge, such as one’s name or date of birth and might be preserved, relearned or updated despite blocked access to EAM, and even in the presence of certain impairments of semantic knowledge of impersonal facts. This may explain why patients with EAM impairments, but an intact autobiographical-semantic memory, might be able to preserve a sense of personal identity [19]. 

Among memory systems, the EAM system develops ontogenetically latest [36,41,42], and is susceptible to neuronal alterations [36] as well as several “sins” [43], including distortions, misinformation and suggestibility. Freud noted that “the material present in the form of memory traces [is] subjected from time to time to a rearrangement” [15] (p. 207). Furthermore, he remarked that there is in general no guarantee for the correctness of our memory; nevertheless, we much more frequently than is justified assume that we can trust its information [44]. Later research on false memory syndromes provided objective support for Freud’s observations. In a combined behavioral and neuroimaging study, it was found that students made roughly 45% errors when judging whether a shown scene had or had not been included in two short movies [45]. These results were confirmed in the same study by the findings of functional brain changes on imaging investigations with functional magnetic resonance techniques. In a recent study, Borsutzky *et al.* [46] pointed to the fact that the “sin” of memory falsification is not limited to memory systems that involve explicit management of information, but can also occur in lower memory systems, such as procedural memory. 

## 4. Memory and the Brain

The quest for an empirically informed theoretical framework that would explain mnemonic processing at the brain level has been characterized by back and forth shifts in interest in theories that promoted a strict mosaic-like localization within specific brain structures and theories that embraced a widespread, Gestalt-like representation within the brain [32]. Nowadays, the theoretical approach to mnemonic processing is a reflection of the neuroscientific stance that both specialization and integration characterize the human brain [47]. It is assumed that information enters the brain via the sensory organs and is then further processed depending on the kind of information and the process selected or triggered. Somewhat simplified, this means that subconsciously processed information predominantly engages unimodal neocortical structures (priming), or—for procedural learning—the basal ganglia, premotor and other motor-related areas. Consciously processed information recruits more widespread networks, which are still largely neocortical for perceptual learning, but include limbic regions for the other two memory systems—the semantic and EAM systems. These two memory systems require the activation of limbic structures where its biological and social relevance is extracted and where the information is compared with already existing, related memories and later bound to and integrated with these (synchronization). Further consolidation occurs during sleep [48], but may extend to years [49]. Storage of memory is largely a matter of the cerebral cortex, although it has to be emphasized that storage is never final, as new information or the retrieval of already existent information leads to re-consolidation and new storage in the context of the last re-consolidation [50,51]. 

Retrieving facts and events requires an engagement of three closely interacting networks, namely activating brainstem structures comprising portions of the reticular activating system, a neocortical network containing the main information of the respective fact or event, plus a limbic network providing the emotional tagging of events or episodes. Importantly, while encoding is based on a hierarchical arrangement of memory systems from procedural to EAM, retrieval allows independence in such a way that no matter how the information was encoded, it can be retrieved in any memory system. Tulving [38] termed this flexibility the SPI-model, SPI referring to SERIAL encoding, PARALLEL processing and INDEPENDENT retrieval. Specific autobiographical details might, for example, be retrieved in the absence of episodic-re-experiencing (recollection) through the autobiographical semantic memory system [19]. Patients with amnesia might still be able to ride a bike, albeit they might not know that they could do so, or might not be able to provide a so-called procedural discourse—verbalizing knowledge about the steps involved in a particular complex motor skill [27,52]. 

There are numerous examples of painstaking analyses in single patients demonstrating that regions of the limbic system are of crucial importance for encoding EAMs. The region that has received by far the most attention, due to the many influential papers on the amnesic patient H.M., is the hippocampal formation. H.M. was a young man who, due to pharmacologically-resistant epileptic attacks, underwent bilateral surgery of his medial temporal lobes in 1953. After surgery, he became severely anterogradely and in part retrogradely amnesic and remained so over many decades until his death in 2008. Many scientists concluded that the region within the medial temporal lobes, responsible for the amnesic condition with respect to EAM, was the hippocampus proper (e.g., [53]).

Two other regional complexes within the limbic system were regarded to be important “bottleneck” structures [54] for EAM encoding. One is the medial and anterior diencephalon, the second is the amygdaloid body. Bilateral damage to the medial—and to a somewhat lesser degree also to anterior—diencephalic structures regularly leads to severe anterograde amnesia [28,55]. As this region contains a number of fibers, a “disconnection syndrome” may underlie the amnesia, as the interaction between distant brain structures may be disrupted. The case of a professor with above-average intelligence illustrates this configuration of damage [56]. After diencephalic damage, he became totally anterogradely amnesic while his retrograde memory was partially preserved (especially for episodes from the first decades of his life). His anterograde amnesia was also accompanied by a significant impairment of his ability to reflect upon his condition, which the authors causally linked to the unusual depth of his anterograde amnesia. When asked, “How would you describe your memory?” he responded, “Well, it is normal.” And when the examiner insisted to ask whether there were no problems, he responded that he would forget his jokes and dreams quite readily. Test results and a conversation with him however immediately demonstrated that his memory for new events was in the range of seconds only. On the other hand, he was able to acquire the skill of reading words, written in a mirror-image manner, and he also showed priming learning, as tested with an incomplete pictures test (see below).

The amygdala, which is situated within the medial temporal lobe and at the same time is part of the basolateral limbic circuit with connections to the diencephalon and the basal forebrain, has been identified as a neural correlate of several memory systems. It was found to be involved in (affective) priming, procedural memory, EAM and even semantic memory (for a review, see reference [57]). The amygdala has also been associated with different phases of memory processing (encoding, consolidation and retrieval) as well as different levels and modalities of processing. The amygdaloid complex represents a major hub for channeling sensory information of biological or social-personal significance to storage in networks located primarily in neocortical areas. With respect to memory, this function implies that the amygdala appraises (automatically or consciously) the salience of newly incoming information and extracts those portions which appear worth being remembered. This holds true for both negatively or positively valenced memories. 

The debate surrounding the role played by the amygdala in amnesic disorder has been perpetuated by the fact that the amygdaloid body is rarely damaged exclusively and bilaterally. An exception is the Urbach-Wiethe disease, an autosomal-recessive genetic condition that can lead to a selective calcification of both amygdalae [58]. As a consequence of bilateral damage of amygdala-structures which are involved in the appraisal of incoming stimuli according to their biological and social significance, patients with this kind of damage may suffer problems with the processing of emotionally-laden EAMs [59,60].

As outlined above, the structures of the limbic system are engaged in a complementary but closely interwoven manner in the acquisition of EAM information, while neocortical, and in part limbic areas represent the major storage places. The combined activation of right-hemispheric fronto-temporal regions serve as trigger stations for retrieving stored EAM events [61,62,63,64]. The corresponding regional complex in the left hemisphere seems to trigger semantic old memories [65].

The fiber system, interconnecting the fronto-temporal regions, is the uncinate fascicle (UF), which has a temporal, frontal and insular part and was ascribed functions in memory and emotional processing [66]. The ventromedial portion of the uncinate fascicle connects primarily the amygdala and uncus with the gyrus rectus and the subcallosal area [67]. According to a histopathological study, in the right hemisphere the uncinate fascicle contains 33% more fibers and is 27% larger than in the left hemisphere [68]. The ventral portion of the right uncinate fascicle has been implicated in the retrieval of EAMs, in particular in ecphorizing affect-laden personal events. 

## 5. Pathophysiology and Possible Neurobiological Mechanisms in Dissociative Amnesia

There are several diseases which could be viewed as falling within the twilight zone of neurology and psychiatry, in particular transient global amnesia (a severe memory impairment usually lasting less than one day) and dissociative amnesia. Both amnesic conditions may be triggered by emotional or physical factors or a combination of the two [28]. Especially for the dissociative amnesia, the underlying causal and maintaining mechanisms remain a riddle, despite several theoretical explanatory frameworks that have been put forth [28,69,70,71,72,73]. One of them emphasizes the role of stressful and psychotraumatic events in the pathogeny of dissociative amnesia; a second theoretical stance underlines the role of executive and attentional dysfunctions and/or memory suppression (motivated forgetting), and a third theoretical model focuses on sociocognitive changes. Furthermore, the possibility of feigning always has to be taken into consideration [28].

The model of Markowitsch [74] posits that the memory impairment in the episodic-autobiographical domain in dissociative amnesia reflects a stress hormone-triggered and -mediated memory blockade, underpinned by a desynchronization between a frontal lobe system, important for autonoetic consciousness, and a temporo-amygdalar system, important for emotional processing and colorization [74]. This memory impairment is opined to be precipitated by adverse life conditions, usually with onset in childhood or early adulthood, and to be modulated by a gamut of factors, such as genes, personality characteristics, medical and psychiatric comorbidities, familial, ecological and cultural environment and epigenetic mechanisms [3]. The massive acute or chronic cumulative stress accompanying these conditions elicits the release of several stress hormones [75], potentially culminating in a nerotoxic cascade. These hormones might bind to the amygdala and the hippocampus—areas with a high density of glucocorticod receptors and roles during encoding and retrieval of episodic-autobiographical events. Depending on the brain developmental window and genetic dispositions, this process may then initiate changes of the morphology or functional connectivity of the above-mentioned structures, which may immediately or after a latency period lead to severe and persisting EAM impairments. In fact, the model of Markowitsch reflects Janet’s [76] view of dissociation and has received empirical support from various neurobiological and neuroimaging studies. The model of Kopelman [77] proposes that the inability to retrieve personal events in psychogenic (dissociative) amnesia reflects an increase in the activity of inhibitory regions of the prefrontal cortex, coupled with a subsequent decrease in the activity of the hippocampus, similar to the one that occurs in suppression or motivated forgetting [78]. In line with Kopelman’s view, Fujiwara and Markowitsch [79] argued that executive control—or the supervisory attentional system [80]—is engaged in holding unwanted or stressful memories out of self-awareness or autonoetic consciousness. This may lead to a kind of overload of the executive system and may reduce frontal lobe abilities necessary for successful retrieval of other non-stressful autobiographical material in dissociative amnesia. Empirical support for the hypothesis that executive functions may co-vary with successful retrieval of autobiographical-episodic memories comes from a study of 14 patients with dissociative amnesia [81]. In this study, deficits in executive function were found in four patients, of whom three performed more than two standard deviations below the control mean. These patients also had more pronounced retrograde memory deficits than those with normal executive functioning.

## 6. Autonoetic and Noetic Mnemonic Processing in Dissociative Amnesia

The most common forms of dissociative amnesia involve retrograde EAM impairments. This is also reflected in the diagnostic criteria of DSM-IV-TR for dissociative amnesia, where the latter is characterized by the inability to recall personal events, usually of a traumatic nature. DSM-IV-TR [26] distinguishes between localized amnesia, selective amnesia, generalized amnesia, continuous amnesia and systematized amnesia [69], while in the psychological literature several other forms of amnesia are found as well [70]. A particular form of retrograde dissociative (psychogenic) amnesia has been coined the “mnestic block syndrome” [71,72]. This latter term was first introduced by Markowitsch [72,73], and then employed by other investigators as well (e.g., [82]). 

The mnestic block syndrome is characterized by an EAM block and—in most instances—a preservation (or a relatively quick regain) of semantic memory and mnemonic processing in other “lower” memory systems [83]. The patients are able to read, write, calculate, and largely know how to behave in social situations. They are able to acquire and store new memories long-term; frequently, however, their new EAMs are less emotionally-tagged than in normal subjects [30,84]. These patients often display a strikingly decreased concern about their impaired state than their immediate partners or relatives, a phenomenon already described by Janet [76] as *“la belle indifference”* (*cf.* also reference [85]). Sometimes, the memory impairment might encompass semantic material (such as autobiographical-semantic or general knowledge) and even non-declarative (procedural) forms of memory [69,86,87]. The semantic or procedural memory impairments are usually transient in nature, though in some cases long-lasting impairments have been reported. Not infrequently, the mnestic block syndrome may occur after minor accidents, such as mild head injuries [3,69,88]. The syndrome is frequently diagnosed in young adults, such as in the third and fourth decade of life [89,90,91]. This age of distribution, on one hand, echoes the negative correlation found between age and dissociation scale scores. On the other hand, it may reflect developmental differences in windows of vulnerability to stress of the main brain structures involved in EAM processes. Another possible explanation is that memory impairments in the elderly are typically attributed to medical illnesses, medication side effects or other psychiatric disorders. Furthermore, new research data show that mechanisms that capitalize on memory suppression become more difficult with age [92]. Dissociative amnesia might co-exist with other psychiatric conditions, such as major depressive disorder, bulimia nervosa, somatoform disorders and certain personality disorders [93]. Headaches and sleep disturbances have also been reported [89]. Loewenstein [94], for example, remarked that dissociated memories in psychogenic amnesia can often reveal their presence in nightmares. Kritchevsky *et al.* [95] also described the case of a man with severe retrograde functional amnesia, whose EAM recovery was accompanied by nightmares with content involving past personal traumatic experiences. Although a connection between REM sleep and consolidation of emotional memory has been put forth by several authors, it has, however, not been unequivocally substantiated empirically (for a review see reference [96]). 

Some patients with functional or dissociative amnesia perform within normal limits on laboratory tasks that tap into Theory of Mind abilities. Others, however, encounter significant difficulties with judging the feelings and intentions of others [91] after the onset of memory “loss”. Although the neural underpinnings of these changes are still largely unknown [9], one study reported that severe EAM deficits in patients with various neurological conditions were found to affect the updating of moral character judgments and therefore influence the way these patients perceive and behave towards others [97].

In the context of a blocked access to past events, patients with psychogenic or organic retrograde amnesia are often unable to plan for their personal future, being imprisoned or trapped in an extended or forever “noetic” present [98]. Additional changes may occur in the form of changes in eating preferences, smoking or drinking habits or other previously rewarding activities (such as car driving) after the onset of psychogenic amnesia [3]. We conjectured that these changes may partly indicate the fact that several brain structures involved in episodic-autobiographical memory processing have also been reported to be engaged in reward-related processing, decision-making and future-minded choice behavior [3,54,61].

Retrograde dissociative amnesia might at times be accompanied by (a) suddenly leaving the customary environment—home and city—and (b) compromised knowledge about personal identity. In this situation, the condition is listed in international classifications of diseases as dissociative fugue [99]. A century ago, this condition was named *Wanderlust* in Germany; *cf.* e.g., reference [100] and was frequently erroneously attributed to epilepsy (e.g., [100]). Fugues were opined to have a higher frequency during times of war and be more frequent among men, although some studies conducted in non-war periods found no evidence of significant gender differences. Fugue states were described in young adults as well as children [101,102]. Precipitants described included rape, sexual assault, combat, marital distress, financial problems or legal difficulties [93]. Most fugues were not found to entail the formation of a new identity. Though they were usually reported to be transient and fully reversible, some prolonged courses were also described [103].

As mentioned above, various degrees of anterograde memory deficits could, however, accompany retrograde functional amnesia. The term anterograde amnesia was reportedly advanced by Charcot [104] to account for the pathological forgetting of events that happened after the traumatic event. In some cases of probable isolated functional retrograde amnesia for personal events following a neurological triggering event, anterograde amnesia might be present initially, but then it may resolve or become subtle [30]. Markowitsch and co-workers, as well as other researchers, observed that although a substantial number of patients with severe retrograde amnesic conditions might still be able to acquire new memories for long-term storage, these anterograde memories might lack the accompanying first person autonoetic emotional engagement and connection. The above changes in anterograde mnemonic processing could, however, escape capture by standard anterograde memory tests. Levine *et al.* [30] reported a case of isolated dense retrograde autobiographical-episodic amnesia covering the entire life, which occurred after a severe traumatic brain injury, and was associated with a focal lesion of the frontal portion of the right uncinate fascicle. Despite normal performance on standard anterograde memory tests, the patient reported a feeling of disconnection from the post-accident autobiographical events. Subsequent refined testing of his anterograde EAM revealed that he assigned significantly less “remember” ratings to his post-injury autobiographical events in comparison to normal subjects. He also generated less event-specific details from anterograde EAM than normal probands, but this was not statistically significant. 

In comparison to dissociative retrograde amnesia, cases of dissociative anterograde amnesia (inability to store new EAM episodes long-term) with preserved retrograde EAM [3,86,104,105,106,107] or cases of dissociative amnesia with anterograde memory impairments out of proportion in comparison to the retrograde memory impairments have less frequently been reported or acknowledged [108,109,110]. In contrast to the DSM-IV-TR [26] diagnostic description that promotes the view of dissociative amnesia as being retrograde, Janet described several patients with anterograde amnesia that involved a mechanism of dissociation (“psychological disintegration”). He observed that in these cases “instead of losing engrams that they have acquired, patients do not acquire any engram” [110] (p. 485). One of these patients was Mrs. D, a 34-year old woman who was studied both by Janet [110] and Souques [111] at the invitation of Charcot. After suffering an emotional traumatic event, Mrs. D developed retrograde amnesia spanning a period of almost 4 months prior to the incident as well as profound anterograde amnesia. According to Janet, she “could speak well, reason correctly, had kept everything she had learned before, but was forgetting all new facts presented to her after two or three minutes”. 

Several historical cases that had been labeled as Korsakoff’s syndrome would nowadays better fit criteria for a diagnosis of functional amnesia [32]. Stevens [112] described a 44-year old woman who in the context of an episode of melancholia accompanied by various somatic complaints without a medical identifiable basis developed severe anterograde and retrograde memory problems and a feeling of unreality. The memory and consciousness impairments remitted after several weeks, while the depressive symptoms lagged behind. 

Another interesting historical case that would likely be nowadays subsumed under the category of functional amnesia is the case of a man who after carbon monoxide intoxication developed both anterograde and retrograde amnesia [113,114,115]. The patient's retrograde amnesia followed typically Ribot’s law [116]. He had a good remembrance of events from his youth, but practically no recall of his recent past. Grünthal and Störring [114] speculated on the morphological substrate of his amnesia and negated the existence of diffuse brain damage, but acknowledged the possibility “that the more refined physical-chemical processes of large brain areas might have suffered so differently in their dynamics or quality that especially the correlates of mnestic functions are affected” (p. 368). They preferred, however, to assume that distinct brain portions such as the mammillary bodies might have been damaged.

In 1933, the patient married his fiancée (which had been mentioned already in the 1930 report) and lived at home. He was still markedly amnesic and introduced his wife consistently as his fiancée. He was always happy to see her as if he had just fallen in love. He showed appropriate behavioral manners, such as taking off his hat when entering the church or when being greeted, and was able to behave well during meals and to explain industrial drawings he made about 10 years previously. But he used external aids for memorizing. For instance, he once explained that it must be Sunday because he was wearing a suit or that he would not be traveling on a train, as he was not dressed appropriately. He also helped his wife in climbing a mountain, as he knew from the time before his accident that she had difficulties on such occasions.

In 1999, Markowitsch and co-workers [106] described a case of functional anterograde amnesia in a young woman who suffered a whiplash injury and developed a severe episodic block, which persisted for more than 15 years. After the whiplash injury, she displayed an ongoing inability to acquire any new memories for long-term storage, while her episodic-autobiographical memories up to the time of the incident leading to whiplash injury remained precise and vivid. Similar to cases reported by Kapur *et al.* [117] and O’Connor *et al.* [118] this young woman manifested accelerated forgetting, while storing information successfully for one to four hours. Recently, Smith *et al.* [86] also described the case of a woman with anterograde functional amnesia who had a long-term memory span of roughly four hours (see below).

## 7. Familiarity in Dissociative Amnesia

Recollection entails recalling associative elements of knowledge or specific contextual details of a previous event so that one can re-experience or relive the past. Familiarity requires memory decisions based on matching processes between retrieval cues and memory representations, in the absence of recollective experience [28]. Another view of familiarity is a “fast, unintentional retrieval process in which memories lack contextual detail” [119] (p. 177). Debates continue to surround the differentiation between familiarity and recollection, with respect to neural correlates and type of management of information [28,119]. At least some of the findings on dissociations with respect to neural correlates between recollection and familiarity-based recognition were suggested, in fact, to reflect differences in memory strength [119]. Some authors view familiarity as bearing a connection with the (noetic) perceptual memory [36]; others view familiarity as having overlaps with conceptual priming. Furthermore, there are suggestions that conceptual priming may involve an explicit form of management (retrieval) of information [119]. We propose conceptualizing familiarity as part of a spectrum and having an objective and subjective component. At one pole of the spectrum, there is familiarity-based recognition to which perceptual memory is intimately linked. At the other pole there is the familiarity linked to anoetic memory systems (priming). In the absence of an acknowledged feeling of familiarity, objective evidence for familiarity may be inferred from behavioral and/or physiological studies. The use of skin conductance response (SCR) has, for example, demonstrated heightened electrodermal responses to items or faces of personal salience in dissociative amnesia [120,121] or prosopagnosia [122,123]. A review of our own cases and other cases reported in the literature suggests the existence of non-uniformity with respect to the preservation of the familiarity-based recognition and feelings of familiarity in patients with dissociative amnesia. Kohnstamm [124] described a case of both retrograde and anterograde, suggestive of a functional amnestic condition. This was a former soldier who had been buried alive, but had no physical complaints. The patient’s short-term memory was normal; however, he did not remember dates from history or geography, which he had learned in school. His musical repertoire, however, was still fully existent; while he was not aware of the musical titles, he was able to play them accurately. Similarly, he recognized his closest relatives, but not his more distant friends, although he still was aware of the fact that he had known them. 

Kohnstamm [125] (p. 375) concluded that the “quality of familiarity” had survived in the patient. The patient, however, had severe anterograde amnesia as well (but no trace of confabulation). Kohnstamm emphasized that the patient’s remembering was greatly influenced (*i.e.*, facilitated) by emotional stimuli and when he was interested and engaged in a topic (p. 378). He remarked that this observation might be used pedagogically and for memory training. Here again, as in other studies of this time, similarities are apparent to the presently discussed division of memory into episodic, semantic, and non-declarative memories. Kohnstamm stated that his “knowledge of language, calculation, and requirements for daily living” (p. 380) was preserved. 

Markowitsch, Fink, Thöne, Kessler and Heiss [99] described patient N.N., who suffered an episode of fugue with loss of personal identity and complete blocked access to his past. During his fugue episode, he rode his bike for long hours. When he stopped and watched his face in the windows of different stores, he reportedly had difficulties recognizing himself. Impaired self-face recognition has been reported in other cases of psychogenic amnesia [126] and we speculated that it may reflect a right hemispheric dysfunction often encountered in dissociative amnesia [127]. When his wife came to visit N.N. in the hospital, he did not recognize her and thought that the doctors were trying to couple him with a woman, unknown to him. In spite of judging his wife as “alien” to him, he nevertheless returned with her to their family home. 

The case described by Markowitsch and colleagues shares some similarity to the one presented by Kanzer [90]. This was a 25-year-old woman who suddenly lost access to the episodic-autobiographical material from her last five years. When her husband arrived at the hospital to take her home, she consented to go with him, in spite of saying that she did not recognize him, stating that: “He must be my husband because I heard him mention my brother’s name to you” [90] (p. 715). Commenting on other cases of dissociative (psychogenic) amnesia, Kanzer [90] noted that sometimes memory arises when a relative returns. In other cases, the patient shows no evidence of recognition, but has, as illustrated above, no objection to being discharged from the hospital home in the custody of a “stranger”. One could argue that the patients’ willingness to return home in the company of a person that they denied recognizing might stem from the familiarity fuelled by a still intact anoetic priming memory system. Another explanation may have to do with a higher suggestibility that has been described in the patients with dissociative amnesia [93]. Another possible explanatory avenue is a shift in the level of consciousness associated with the mode retrieval coupled with a lack of acknowledgment of that shift [87,128]. This shift is illustrated by the confession that patient FF later made to the researchers: “I was aware of knowing German in written and spoken form somehow after about 10 days…. It was a part of my life I just wanted to lock away in a dark chamber. I can’t even say if it was an active will or passive defense…. The point where this disorientation was replaced by neglecting the truth is not easy to find and somehow undefined…. In the last test, the lie detector, some of the things were common (familiar) and my mother’s name went through my ‘personal barrier’.” [87] (p. 1146). 

## 8. Conditioning and Priming in Dissociative Amnesia

The traditional view of dissociative (psychogenic) amnesia posits that implicit processing of information, such as that coupled with classical conditioning, priming and procedural memory is preserved in this condition. This view is consistent with the one from neurologically-linked forms of amnesia. The famous anecdotal report with respect to classical conditioning is that of Claparede [128] who one day shook the hand of an amnesic female patient while hiding a pin in his hand. The next day the same patient refused to shake hands with the Swiss neuro-psychiatrist, although she was not able to recall the reason. 

The strong nature of aversive classical conditioning *versus* perhaps the appetitive one is illustrated by de Renzi *et al.* [27] in their description of Andrea (a fictitious name), a 58 years old man with a two-year history of purely retrograde functional amnesia. Despite having difficulties with recognizing food by sight and taste, Andrea continued to dislike coffee and tomatoes in the same way he had done before. The opposite happened however in the patient with sudden onset of retrograde amnesia and loss of identity that was described by Thomas-Antérion and colleagues in 2008 [129], who despite having been a heavy cigarette smoker before the onset of the amnesia, totally quit smoking immediately afterwards. Since the appearance of the first studies on priming, it was generally assumed that priming is preserved in patients with anterograde or retrograde amnesia [130,131]. Squire *et al.* [132] stated, “that priming is fully independent of declarative memory” (p. 252). Severely amnesic neurological patients were reported to have comparable performance with controls when asked to visually identify a degraded variant of words or pictures they had encountered before, despite having no capacity for recalling or recognizing them [133]. The studying of patient H.M. was instrumental in providing the grounding framework for the investigation of implicit and explicit management of information in memory research. The assessment of H.M. revealed that he could still acquire information on the levels of conditioning, priming, and procedural memory. Furthermore, he remained able to reflect upon his condition and even upon his emotions/feelings, despite having undergone bilateral removal of the amygdala in addition to removal of the parahippocampal-entorhinal cortex, and the anterior hippocampus, as suggested by the following words attributed to him: “Every day is alone what enjoyment I’ve had, and whatever sorrow I’ve had.”

The burgeoning interest in priming and other implicit forms of memory management ignited by the study of H.M. and other patients with mediotemporal lobe amnesia has been paralleled by increased sophistication and variety in testing paradigms. The employment of new testing methods, however, has recently revealed a less clear-cut and homogeneous picture of priming in patients with amnesia resulting from mediotemporal lobe damage. Several studies found intact repetition priming, but impaired relational priming, supporting the view that a main function of the hippocampus is to bind features of an event or item or relations between items together rather than consciousness *per se* [10,119]. Azienza *et al.* [134] found a decreased semantic priming effect in patients with mild cognitive impairments compared to normal elderly persons (conceptual priming). Other authors, such as Katharina Henke [135] stated that the hippocampal system—usually opined to participate exclusively in conscious (noetic, autonoetic; *cf.* reference [27]) forms of memory—might be involved in priming as well (and not only in EAM and semantic memory). Furthermore, she proposed a classification of memory systems independent of the criterion of consciousness, and solely based on processing modes or processing operations. In the light of evidence from various other sources—reviewed recently in Dew and Cabeza [119]—it has increasingly been acknowledged that certain forms of priming, in particular conceptual priming, are at least in part affected by impairments of the semantic and the EAM systems. Consequently, it is not surprising that there is evidence of deficient performance in certain priming tasks in patients with a background of functional or dissociative amnesia as well. On the other hand, in a substantial number of patients with dissociative amnesia, the performance of various tasks that tap on the priming and procedural memory systems remains largely preserved. This allows these patients to maintain their ties with their social and biological environment and ground a sense of self-coherence.

The work of Markowitsch and colleagues in patients with neurological or dissociative types of amnesia or episodic-autobiographical memory impairments has also pointed out a non-homogeneity of findings with respect to priming and procedural memory, and has suggested several ways in which explicit and implicit management of information may interact at both the behavioral and neural levels [135].

In a study that compared 15 brain-damaged patients (mostly with right hemisphere damage) with 15 patients after non-neurological surgical interventions, perceptual picture priming test performance was lower in patients with brain damage, while verbal priming did not differ among the groups [135]. The verbal (word and idiom-sentence priming) priming was not different between the groups. A direct relationship between WMS-revised General Memory Index (MQ) and to a lesser degree also between IQ and verbal priming performance was found. Incidentally, in a recent pilot study, the magnitude of the priming performance, as assessed by a word-stem completion test, was greater among participants who achieved verbal memory scores higher than 110 on Wechsler Memory Scale-Revised in comparison to “normal memorizers” [136]. 

In the case of the above-mentioned 67-year-old professor with profound anterograde amnesia and more selective retrograde amnesia, Markowitsch and co-workers employed several testing paradigms tapping into priming, such as Gollin’s Incomplete Pictures Test [137], Word Stem Completion, Sentence Completion, Phrase Completion, and procedural memory system (Mirror Image Reading). The obtained findings pointed to a non-uniformity of performance levels in these tasks. Overall, the patient’s performance in the Gollin’s Incomplete Pictures Test was quite poor in comparison to controls, but significantly improved from session to session, in the absence of any evidence for explicit memory for the test items, although his performance did not reach the level attained by the controls. Word stem completion brought ambiguous results that were attributed to the special adherence of the patient to the German language. Both the very poor starting level in identifying the incomplete pictures and the patient’s failure to acquire any non-idiom words over three sessions in the sentence completion test were attributed to his profound amnesia that might have impacted on his ability to combine or reformulate time-spaced information. Another posited interpretation for the patient’s difficulty with sentence completion test was that he was able to process re-(learned) known idioms, but failed to acquire any newly presented ones, due to his massive anterograde amnesia. Overall, the results pointed to several aspects concerning processing in memory systems. The improvement in performance in the incomplete picture test supported the idea of the existence of dissociable neural substrates for episodic and (perceptual) priming memory systems. The mixed results with respect to performance involving other priming testing paradigms is in conformity with the view that dissociating between implicit and explicit forms of information management might pose a bigger challenge for priming testing paradigms other than the ones investigating the priming of pure perceptual information. These testing paradigms might be facilitated or supported by performance on other memory systems, reflecting the “porous boundaries” or dynamic interaction that exists between diverse memory systems at both the behavioral and neural levels [119].

With respect to dissociative amnesia, Markowitsch *et al.* [72,74] described the case of A.M.N., who was a 23 years-old employee of an insurance company with 11 years of education. After discovering one evening the outbreak of a fire in the cellar of his house, he immediately left the house shouting “Fire, fire,” while his friend—who was in the house at the time as well—called the fire department, who immediately extinguished the fire. In the night of the event A.M.N. and his friend retired to bed as usual, but the next morning, upon waking up, A.M.N. thought that he was only 17 years old, did not remember any personal events beyond this age and also became unable to acquire new events long-term. Three weeks after he was admitted to an university clinic, where he underwent medical and laboratory work up (including conventional structural brain imaging), which yielded unremarkable results. On neuropsychological examination, A.M.N. showed memory deficits in several memory fields, including poor performance in comparison to normals on perceptual priming tests (incomplete picture test). He also showed poor short-term memory, problems in word finding, reading comprehension and calculation. After three weeks of psychotherapeutic interventions in the hospital, the patient recollected one of his childhood memories. He remembered that at age 4 he saw a road accident that involved two cars crashing, one of which burst into flames. He witnessed the driver’s death in the fire, his screams as he pressed his head against the glass and hammered at the window. This memory was confirmed by the patient’s mother, who witnessed the event as well. Since then, an open fire was reportedly perceived as life-threatening by him. The authors hypothesized that the witnessing of the traumatic incident at age 4 already initiated subtle biological changes and that the latter witnessing of the fire outbreak in the house triggered a magnified biological response in the form of a cascade-like release of stress hormones, such as glucocorticoids [75], which led to the mnestic blockade that covered the last 6 years of his life. Interestingly, A.M.N.’s inaccessibility of his former memories was accompanied by an overall reduction in his cerebral glucose metabolism (as measured by fluorodeoxyglucose—FDG-PET), together with an even more pronounced reduction of the metabolism in the memory-sensitive regions of the temporal and diencephalic areas [73]. The hippocampal formation of A.M.N. was found to be bilaterally hypometabolic. A metabolism of three standard deviations below that of normals was found in his right temporo-basal, left temporo-mesial, left and right fronto-basal, left insular and dorso-parietal cortex. A metabolism of two standard deviations below normal was found in the left temporo-basal, the right and left fronto-mesial, the right insular, the left cerebellum and the left putamen. However, treating the patient psychopharmaceutically (with antidepressants) and psychotherapeutically resulted in a principal reinstatement of his old memories and a reinstatement of his ability to encode new episodic events long term.

The fact that the blockade of his conscious memories for personal events spanning the last 6 years of his life may be accounted for by several experiences that occurred during that time, which likely had an intense and negative emotional connotation: he disclosed his sexual preferences to his parents and his circle of friends; he experienced disagreements with his parents and ended up leaving both his school and the family home.

Another interesting case with a variant of dissociative amnesia (dissociative fugue) described by Markowitsch *et al.* [99] was already briefly mentioned above. He was a 37-year-old man with low self-esteem and a history of maltreatment by his parents. He grew up with a mother who was very critical of him. Later on, he married a woman who apparently resembled his mother. Reportedly, his wife decided one day that they should leave for a vacation the following week. 

Three days prior to the intended family holidays, N.N. was supposed to get some bread rolls from the bakery for breakfast. He took his bicycle, but instead of returning home, he continued biking for a number of days, from North to South Germany, until he reached a large city. At the central station, a person from the Salvation Army suggested to him to enter the city’s psychiatric university hospital, which he actually did. 

In the clinic, N.N. felt quite happy, made friends and appeared not to care about his condition of having no access to his past (“*la belle indifference*”). After a police search, N.N. was found and visited by his wife. Contrary to the belief that states that confrontation with reality leads to immediate reinstatement of memories—this did not happen to him. He did not recognize his wife, but nevertheless returned to their marital home—complaining however on their arrival about the furniture and curtains. He also failed to recognize his children. Interestingly, he changed his habits (he stopped driving) and his job. He ceased having allergic and asthmatic attacks, and gained 15 kg, in spite of denying feeling hungry and saying that no food tasted particularly good to him any more. He was able to re-learn his past and retrieve portions of it in a neutral, unaffected manner. Other cognitive functions were largely spared or regained within weeks.

N.N. was tested neuropsychologically and with functional imaging. Water-PET was used in combination with the design of Fink *et al.* [62], during which the patient was confronted with events from his personal past. While the normal probands had predominantly right temporo-frontal activation [62], N.N. had a left-hemispheric activation of these regions. In light of other data on brain activations during memory retrieval (e.g., [138,139]), this finding was interpreted as suggesting that the patient perceived his own episodic-autobiographical episodes as if they were belonging to a third, neutral person. 

N.N.’s perceptual priming, tested with the incomplete picture task [137], was at the second presentation somewhat lower than that of students, average age 24 years, tested with a 24 h delay. 

In a patient with psychogenic amnesia, Kopelman *et al.* [140] reported no evidence of word-stem completion priming for given names and names of places from a patient’s personal past, albeit his performance for the same information on a forced recognition task was above mere chance. Following the procedure of Kopelman *et al.* [140], Markowitsch *et al.* [99] constructed a Word-stem Completion of Autobiographical Memory task for names and places from the patient’s past. The test consisted of 14 names of people and 5 names of companies. The 19 word-stems were presented together with 40 distractors. The first 10 word-stems on the list were distracters. The patient was asked to complete the word-stems with the first name that came to his mind. While working on the task, N.N. came to the word-stem “TAR” (used for Tareck, the name of his son, which is uncommon in Germany). He asked if the test was constructed especially for him because the three letters could have been taken from the name of his son. After realizing that this was the case, he changed his working strategy and he scanned the other word-stems and filled in the names which he in fact recognized from his past. He completed 5 out of 19 names with names from his personal history. In addition, he completed three of the baseline items with names from his past. In this case, the results of the test were not interpreted as a measure of implicit memory as the spontaneous awareness of the test design prompted the patient to adopt a novel non-incidental strategy toward completing the task. 

Serra *et al.* [69] described normal repetition priming in a Stem Completion test [141] in a 34-year-old woman with a history of retrograde psychogenic amnesia with loss of personal identity who was investigated 8 years later for persistence of retrograde memory impairments, in spite of recovery of “isles” of autobiographical events. Patient F.F., described by Glisky *et al.* [87], also performed within normal limits on an implicit Word-Stem Completion test [121]. 

## 9. Procedural Memory and Dissociative Amnesia

Freud viewed repeating, remembering and working through as three stages of a continuous process [142]. As Russell [143] pointed out we are creatures of habit by design, and so-called repetition compulsion is education-resistant. Lower memory systems, such as conditioning, priming and procedural memory are not only education-resistant, but seem also less vulnerable to insults. Data from patients with severe amnesia due to medial temporal lobe damage suggest that they are quite capable of learning and remembering motor skills. A study that looked at nine patients with chronic stable amnesia with bilateral medial temporal lobe damage due to herpes simplex encephalitis or anoxia, and one with thalamic stroke and 25 matching normal comparison subjects, emphasized preserved ability for the acquisition of a variety skills relevant to real-world activities in patients with amnesia, and proposed that procedural memory training should be an integral component of comprehensive rehabilitation programs for patients with memory impairments [52]. 

Not infrequently, patients with dissociative amnesia report being unable to perform tasks that engage the procedural memory system. Several patients with dissociative or functional amnesia have reported difficulties with procedures that they had been competent or proficient at before the onset of amnesia, such as word finding, writing, reading and reading comprehension, and speaking the native language or a second language, cooking, ironing, driving, riding a bike, tying shoes, using a pen, penmanship, using a fax, using a computer, typing, playing the piano, using common tools (razors), knitting, how to tell the time from a watch and calculation [28,69,95,125,127,144,145]. There have also been reports of changing in penmanship in functional or dissociative amnesia, such as adopting a different signature from an earlier younger époque [28,146]. In the paper of Glisky *et al.* [87] a patient with a psychogenic fugue condition is presented, who lost access to his autobiography together with native German language, while his implicit memory and knowledge of German grammar structure remained intact [147].

Markowitsch *et al.* [148] described a patient with retrograde amnesia (probably of mixed etiology) who no longer knew or remembered that he had possessed a precious collection of antique clocks, but was able to manipulate the fine and complicated mechanical components within them without hesitation. Consequently, while he did not consciously remember his skills (serially encoded), he could retrieve them in an automatic way (parallel retrieval of procedural memories).

de Renzi and colleagues [28] subsequently cautioned about drawing conclusions about impairments in procedural memory in cases of functional or dissociative amnesia, for several reasons. They stated that some of the reported skill performance difficulties could not be observed directly, as the patients refused to perform them in front of others or they did not commit themselves to relearning. They also pointed out a possible semantic deficit that may have accounted for the initial awkwardness in the task, as the patients could not recall what specific actions were necessary for the performance of the motor act. In the case of Andrea, de Renzi and colleagues noted that the change in penmanship persisted, in spite of attempts to rehabilitate it, suggesting a true, persistent procedural memory deficit. However, only a few studies have provided objective evidence of procedural memory impairments in functional or dissociative amnesias. Smith *et al.* [68] showed that patient FL performed very poorly on two motor skills tasks (mirror drawing and rotary pursuit task, respectively) during both the first and second days of testing in comparison to controls, and in addition exhibited no improvement from the first day to the second day. The authors speculated that an experience of disability might have created tentativeness, anxiety, and a sense of helplessness, which may have affected the performance. However, the patient BC described by Serra *et al.* [69] showed a clear improvement in mirror reading skills with practice. 

In fact, there are also suggestions that amnesia may, in some cases, favor the performance of highly automatic behaviors. N.N.’s state of altered (autonoetic) consciousness [99] did not interfere with his automatic riding of his bike (“ambulatory automatism”) [94] and may have even enhanced it. Maldonado and Spiegel [93] reported that certain forms of dissociation, such as suspension of critical thought, may enhance the performance of coordinated and complex motor acts in athletes. In contrast, overthinking might disrupt the performance of highly-skilled procedures in proficient athletes [149]. 

## 10. Conclusions

The “remains of the day” in patients with dissociative amnesia are mainly represented by the bulk of their implicit memories which, to a large degree and in a substantial number of patients, continue to be maintained. These memories include habits that are lived and acted [150]. In the presence of an impaired autonoetic consciousness, these memories and habits continue to ground and preserve a sense of self that goes beyond that of a core self. The importance of implicit, automatic management of information for one person’s well being had been emphasized by William James [7], who viewed habits as “the enormous fly-wheel of society, its most precious conservative agent” (p. 132). James argued that “*we must make automatic and habitual, as early as possible, as many useful actions as we can*, and guard against the growing into ways that are likely disadvantageous to us, as we should guard against the plague. The more of the details of our daily life we can hand over to the effortless custody of automatism, the more our higher powers of mind will be set free for their own proper work. There is no more miserable human being that one in whom nothing is habitual but indecision, and for whom the lighting of every cigar, the drinking of every cup, the time of rising and going to bed every day, and the beginning of every bit of work, are subjects of express volitional deliberation” (p. 134). 

The need for a balance between implicit and explicit mnemonic processing is confirmed by the literature on great mnemonists (mentioned above), who often perceived their extraordinary explicit mnemonic processing abilities as being a burden, rather than a gift. The interplay between implicit and explicit management of information associated to various memory systems is, however, quite complex. This is also suggested by the non-uniformity of findings in memory performance in patients with dissociative amnesia. Researchers embarking on the work of disentangling the neural and behavioral correlates of implicit *versus* explicit modes of mnemonic processing seem to be faced with a number of challenges. One of the grand challenges may come from the fact that, as William James stated [7], “consciousness, then, does not appear to itself chopped up in bits…. It is nothing jointed; it flows. A ‘river’ or a ‘stream’ are the metaphors by which it is most naturally described” (p. 145). 
